# NK Cells in Health and Disease

**DOI:** 10.3390/biomedicines13061312

**Published:** 2025-05-27

**Authors:** Matthew D. Blunt

**Affiliations:** Clinical and Experimental Sciences, University of Southampton, Southampton SO16 7YD, UK; m.d.blunt@soton.ac.uk

Natural killer (NK) cells are cytotoxic innate lymphocytes that have a critical role in anti-viral and anti-tumour immunity [1,2,3]. Their activation is tightly controlled by the integration of signals from a wide variety of germline-encoded non-rearranged inhibitory and activating surface receptors. In addition to their direct cytotoxic activity, NK cells also secrete chemokines and cytokines, which can shape adaptive immune responses, for example, through the recruitment of dendritic cells and the enhancement of macrophage and CD8 + T cell activity. Furthermore, via their ability to engage the Fc region of tumour-targeting antibodies, NK cells are thought to contribute to the efficacy of approved monoclonal antibody therapies such as rituximab and obinutuzumab. The adoptive transfer of these cells holds significant potential for the treatment of cancer, with recent clinical trials demonstrating promising efficacy and excellent safety [4]. In addition, the utilization of NK cell therapy for the treatment of autoimmune disorders such as systemic lupus erythematosus and neurological pathologies such as Alzheimer’s disease has gathered interest, with multiple clinical trials in this setting currently underway [5].

Increased knowledge of the role of NK cells in health and disease will allow for the design of improved therapeutic strategies. This Special Issue gathers recent advances in knowledge on NK cell function within the five papers discussed below.

Bacille Calmette–Guerin (BCG) is a vaccine primarily used for protection against tuberculosis; however, it is also approved as a treatment for early-stage bladder cancer. BCG is known to induce broad immune cell activity, including enhanced NK cell function. In their study, Ruiz-Lorente et al. investigated the relevance of the NKG2A: HLA-E immune checkpoint axis in bladder cancer patients when treated with BCG. Their analysis of 325 patients revealed that the HLA-B-21M/T genotype is associated with progression-free survival and overall survival following BCG treatment. Interestingly, the -21M haplotype has previously been shown to support NKG2A+ NK cell function [6], indicating that these cells may play a role in this setting.

There are currently over 100 clinical trials assessing the efficacy of adoptively transferred NK cells against cancer [5], and the source of these cells includes cord blood, peripheral blood from adult healthy donors, and established NK-like cell lines. In their study, Alekseeva et al. compared the activity of peripheral blood-derived NK cells from healthy donors with the commonly used NK-like cell line NK-92 against multiple in vitro tumour spheroid models. The differences they identified have implications both for the interpretation of in vitro studies utilizing NK-92 cells and for their clinical translation. It should be noted that most clinical trials assessing NK cells in the adoptive transfer setting utilize primary ones rather than NK-92 [5], with the latter requiring irradiation prior to infusion to prevent their uncontrolled expansion in patients.

The activation of NK cells is tightly controlled via multiple inhibitory surface receptors, and the immune checkpoint target TIGIT (T cell immunoglobulin and immunoreceptor tyrosine-based inhibition motif domain) is expressed and functional on these cells [7]. Xie et al. investigated the expression and role of TIGIT on NK cells in patients with core binding factor-acute myeloid leukaemia (CBF-AML). They identified that CBF-AML patient-derived TIGIT+ NK cells possessed weaker cytotoxicity against target cells ex vivo compared with TIGIT– NK cells. Furthermore, the authors identified that a higher frequency of TIGIT+ NK cells was significantly associated with shorter relapse-free survival in CBF-AML. Given that TIGIT blockade is currently under evaluation in clinical trials for other types of cancer [8], this study highlights the potential of targeting TIGIT to stimulate anti-cancer immunity in patients with CBF-AML.

Haematopoietic stem cell transplant is an approved treatment for patients with lymphoma, and NK cells are the first lymphocyte population to reconstitute after this procedure, with a higher NK frequency associated with improved outcomes [9]. In their study, Porrata et al. identified that reduced inhibitory KIR2DL2, along with increased activating NKp30-expressing NK cells at day 100 post-transplant, was an independent predictor for overall and progression-free survival in patients with non-Hodgkin lymphoma.

Graham et al. discussed the current knowledge on NK cells’ role within the lymph nodes, a vital tissue site for the generation of adaptive immune responses. NK cells are rapidly recruited to the lymph nodes in response to infections or vaccination and can shape B cell and T cell immunity within lymphoid organs and aid in viral clearance. Furthermore, the lymph nodes are a frequent site of metastasis in cancer patients, and the augmentation of NK cell function at this critical tissue site may be important in the design of improved strategies to eradicate metastatic disease.

Together, these articles help to progress our knowledge of NK cell function in health and disease, and we thank the authors for their contributions. Given their promising efficacy and excellent safety in clinical trials to date, NK cells hold significant potential for the treatment of a variety of diseases. Further research will allow for their optimal utilization, and current key areas of focus include the improvement of NK cell persistence and function within the immunosuppressive tumour microenvironment.
